# Expected population prevalence following decriminalization of recreational use of cannabis in Sweden

**DOI:** 10.1186/s42238-026-00405-z

**Published:** 2026-02-13

**Authors:** Filip Andersson, Mats Ramstedt, Robert Thiesmeier, Cecilia Magnusson, Nicola Orsini, Maria Rosaria Galanti

**Affiliations:** 1 Department of Global Public Health, Karolinska Institutet, Stockholm, 17177 Sweden; 2 Centre for Epidemiology and Community Medicine, Stockholm Health Care District, Stockholm Region, Box 45436, Stockholm, 10431 Sweden; 3 Department of Clinical Neuroscience, Karolinska Institutet, Stockholm, 17177 Sweden; 4 The Swedish Council for Information on Alcohol and Other Drugs, Östergötagatan 90 11664, Stockholm, Sweden

**Keywords:** Cannabis use, Policy, Decriminalization, Time series, Sweden

## Abstract

**Background:**

Many countries and jurisdictions have recently adopted more lenient cannabis laws. While use remains prohibited in Sweden, this stance is debated due to high drug-related mortality. To inform this debate, we predicted changes in cannabis use following a hypothetical decriminalization in Sweden.

**Methods:**

Jurisdiction-level data on self-reported cannabis use from 12 countries (across Europe and Australia) and four U.S. states were used in a multilevel meta-regression model to predict the most likely changes in past 12-month and past 30-day cannabis use following decriminalization in Sweden.

**Results:**

We predicted an immediate increase in the prevalence of both past 12-month and past 30-day cannabis use following decriminalization. However, the longer-term trends differed between these measures. For past 12-month use, the gap in prevalence between scenarios with and without decriminalization gradually narrowed over time. In contrast, for past 30-day use, the gap widened.

**Conclusions:**

Decriminalizing cannabis use in Sweden would lead to an initial increase of self-reported cannabis use. The extent to which this reflects a genuine increase in use or an increased propensity to disclose the behavior remains to be understood. After this initial increase experimental use tends to stabilize, while recurrent use may continue to increase, probably indicating an increasing pool of individuals transitioning towards dependence. The method we propose for predicting population trends in cannabis use following decriminalization can be easily replicated in other context and used to support evidence-based policy decisions.

**Supplementary Information:**

The online version contains supplementary material available at 10.1186/s42238-026-00405-z.

## Background

The prevalence of cannabis use has increased globally, particularly among young adults, in the past 20 years (Kraus and Nociar 2016). This increase can be interpreted as a change in attitudes towards cannabis use, making it more socially acceptable (Carliner et al. 2017; Felnhofer et al. 2021; Skelton et al. 2022). Parallel with the change in attitudes, changes in legislation have been introduced in several countries implying either legalization or decriminalization of use. These two concepts are closely related, but not identical. Legalization refers to the removal of all penalties related to possession and use of the drug, often also including controlled sales of cannabis (Svrakic et al. 2012; AaD 2023). Decriminalization refers to the removal of criminal sanctions against possession (often under a specified amount) and personal use (Svrakic et al. 2012). After decriminalization, sales of the drug are still prohibited.

Several countries have legalized the recreational use of cannabis, specifically 24 states (and some territorial districts) in the United States (US) (Montgomery and Allen 2023), Canada (Government (n.d.)), Thailand (Kalayasiri et al. 2023), Uruguay (Shulman et al. 2022), Germany (Neu 2023), Luxemburg (Plans 2024), Malta (Plans 2024), Mexico (Plans 2024), South Africa (Plans 2024), one region of Australia (Plans 2024) and Georgia (Plans 2024). Additionally, the Netherlands implemented a legislation that could be classified as a mixture of legalization and decriminalization where purchasing and using cannabis in licensed coffee shops is legal or at least tolerated by legal authorities (Shulman et al. 2022).

Many other countries have recently decriminalized recreational cannabis use, often specifying a maximum amount of possession that is included in this class of usage. Among these countries are Spain (Bąkowski 2024), seven states in the US (Montgomery and Allen 2023), the remaining states of Australia (Shulman et al. 2022), Austria (Bąkowski 2024), Belgium (Bąkowski 2024), Croatia (Bąkowski 2024), Czech Republic (Shulman et al. 2022), Estonia (Bąkowski 2024), Italy (Shulman et al. 2022; Italy Cli 2023), Portugal (Montgomery and Allen 2023), Slovenia (Bąkowski 2024) and Switzerland (Shulman et al. 2022).

Previous meta evaluations of legislation of cannabis use have shown a slight increase in prevalence among young adults following legalization, but no conclusive results following decriminalization (Melchior et al. 2019; Govenment 2021). For example, a study on young adults in Europe did not find any significant change in the prevalence of use following decriminalization (Gabri et al. 2022), and neither did a study of the adult population in five states in the US (Grucza et al. 2018).

At present, the use of cannabis products containing Tetra-Hydro Cannabinoid (THC) is completely prohibited in Sweden (Narkotikastrafflag 1968). Further, the Swedish society has historically endorsed strong anti-drug norms (Narkotikastrafflag 1968; Bejerot 2021). It should be noted that drug policy changes in Sweden typically require parliamentary action through the legislative process. Drug legislation is a central component of Sweden’s national drug policy, aiming to limit the availability and use of illicit drugs including cannabis (Government S 2022). In an international context, Sweden belongs to countries with restrictive drug laws, including criminalization of recreational use (Stenström et al. 2024). Since 1988, drug use has been illegal in Sweden, and in 1993 imprisonment of up to six months was added to the penalty scale for personal use. Extensive enforcement of this legislation has contributed to Sweden reporting the highest number of drug offenses per capita in Europe (EMCDDA 2023). In contrast, surveys report relatively low cannabis use compared to other European countries (Manthey et al. 2021) despite increasing trends since the 2000 s (Montgomery and Allen 2023; Govenment 2021; Shulman et al. 2022). Moreover, a previous study indicated a large underestimation of the use of cannabis in Swedish surveys, at least among young adults (Andersson et al. 2023).

A policy change in Sweden might be motivated by the argument that in Sweden high levels of drug related mortality have been observed compared to rest of Europe (Agardh et al. 2023). This has been interpreted as an increased risk of refraining from seeking care and from disclosing substance-related problems in contacts with the healthcare system in countries with strict drug policies (Røgeberg and Pedersen 2021). Still, recent studies indicate limited support for cannabis decriminalization in Sweden. For example, a 2021 survey found that 67% of adults in the general population believed cannabis use should remain illegal (Sweden TPHAo 2023). This also appears to apply to Swedish political parties, at least prior to the 2022 general election. In a survey of the eight major political parties, only the relatively small Left Party expressed clear support for decriminalization, while the major parties were clearly opposed (Narkotika 2022). It is however possible that this has changed since criminalization of recreational drug use has become increasingly debated and questioned in Sweden in recent years. A recent review by Stenström, Estrada (Stenström et al. 2024) evaluating the impact of sharpened penalty for drug possession introduced in Sweden in 1988 showed that it was not followed by a reduction in the problematic drug use, or by lower drug-related mortality. The authors also concluded that this measure seems ineffective, expensive and even harmful. Furthermore, when the most recent government drug policy inquiry (Sou 2023) did not include an evaluation of this policy, it was criticized by several key organizations, including the Public Health Agency of Sweden and the Swedish Association of Local Authorities and Regions (SKR), which called for such evaluation. On the other hand, one cannot assume specular effects of a more lenient drug policy both positive, i.e. reduced problematic drug use and lower drug related mortality, or negative, i.e. increased problematic drug use and healthcare expenditures. Therefore, the potential implications of such policy change in Sweden need to be understood.

In this study, we aimed to predict potential changes in the population prevalence of cannabis use following a hypothetical decriminalization of recreational cannabis use in Sweden. The study aimed to answer the following research question: Which would be the most likely change in the prevalence of recreational use of cannabis should it be decriminalized in Sweden?

## Methods

All analyses were done using Stata version 18.0 (StataCorp, LP). Ethical scrutiny is not needed because of meta-data.

### Data and design

In reporting on data collection and synthesis we followed the steps suggested by the GATHER statement (Stevens et al. 2016).

First, we collected the following country-level estimates from population surveys: past 12-month and past 30-day cannabis use among individuals of 16 years of age and older; sample sizes; year of decriminalization; and macro level factors. Data was collected from 1994 and onwards depending on availability until 2024. All data was extracted manually and synthesized into one single database in Excel format.

We used five macro level factors, representing aspects of socio-demographic and economic differences among countries, because there are indications that different socio-economic groups may respond differently to policy changes related to cannabis use (Smart and Pacula 2019). The GINI-coefficient (Giorgi and Gigliarano 2017) represents the income inequality within a population. The Socio-demographic index (SDI) (Wang et al. 2020) represents the social and economic development of a country. The share of the population with tertiary education represents the literacy of the country. The share of the population age 15–24 represents the size of the population where cannabis use is most common. Finally, the gross domestic product per capita (GDP) (Stiglitz et al. 2009), represents the average economic asset of the country.

The national level data on share of young adults in the population are categorized as age 15–24 in the original source (United Nations Population Division), while the prevalence of cannabis use is reported from 16 years for most countries.

We used data from North America, Australia, and Europe (12 countries and four U.S. states) because of the stronger similarity of these countries with Sweden in terms of population structures and cultural frames (Fearon 2003). We included data from countries that reported both pre and post decriminalization measurements of prevalence of past 12-month use and past 30-day use. Countries were included if the decriminalization of cannabis use was introduced during the 2000 s and use was not later legalized until the end of study period. Because of this restriction Malta as well as some US-states (Connecticut, Rhode Island, Vermont, and Maryland) were excluded. The include countries and U.S. states are listed in Table 1.

**Table 1:** Included countries and year of decriminalization of cannabis use

**Country**	Year of decriminalization
Australia	2020
Austria	2016
Belgium	2003
Croatia	2013
Czech Republic	2013
Estonia	2005
Italy	2022
Luxemburg	2001
Portugal	2001
Slovenia	2013
Spain	2017
Switzerland	2012
United States	
Hawaii	2020
Louisiana	2021
New Hampshire	2017
North Dakota	2019

To address missing data points on sample size at any given survey, the average between the two closest surveys was calculated. For the other missing data, last estimation carried forward was adopted (Overall et al. 2009). This imputation method was exclusively used for the macro factors, while only observed estimates were used for the prevalence of cannabis use.

In Appendix 1, table A1, we present summarized information on the data used in this paper and the original sources of the data. The synthesized data used in the analysis are provided in Appendix A2 and the analytical codes in Appendix A3.

The pre-registered research plan was published on 2024–12–15 and can be found at https://osf.io/v5pn8/ (Andersson 2024).

### Model definition

The model used to forecast population-based outcomes following a change to a more liberal cannabis legislation in Sweden was divided into three separate steps, the outputs of which were then combined. The three steps consist of: 1) estimation of trends over time of cannabis use in the target population in absence of decriminalization (Baseline), 2) estimation of the likely change in population prevalence following a change in the legislation using interrupted time series design, a method commonly used to study the population’s impact of interventions (Kontopantelis et al. 2015) (Intervention’s effect) and 3) estimation of the effect modification, introduced in the second step, by macro level factors (Influence of macro factor on intervention’s effect). As all data are based on self-reported survey data, the predictions are derived for the expected survey response following decriminalization. For detail description of the three separate steps of the forecast model see Appendix A4.

#### Prediction of cannabis use after decriminalization

We combined the three steps together to define the model predicting the expected cannabis use at a given time following decriminalization as:

$$\begin{aligned} &f\left(country,\:year,\:intervention, \:\right. \\& \left. time\:post\:intervention,\:macro\:factor=X\right)\\&={\alpha }_{c}+{\beta }_{c}*year+{\Delta }_{1}*X*intervention \\& \quad +{\Delta }_{2}*X*time\:post\:intervention \end{aligned}$$

Here, ${\alpha }_{c}$ is the prevalence of cannabis use in the specific country at start of time series, ${\beta }_{c}$ is the average yearly change in the prevalence of cannabis use, ${\Delta }_{1}$ is the initial intervention effect dependent on macro level factor X, and ${\Delta }_{2}$ is the average yearly change after intervention, depending on macro level factor X. For more detail see Appendix A4.

### Parameter estimation

We considered a model composed of two sets of parameters: 1) country specific parameters, and 2) average effect parameters. We then classified these two parameters as random effects (1) and fixed effects (2). To account for the sample sizes from which the observed data was derived (Appendix A2), we defined each observation as a separate study and approached the interrupted time series analysis as a meta-analysis (Korevaar et al. 2020). Therefore, we used multilevel meta regression to estimate the fixed effects ${\Delta }_{1}$ and ${\Delta }_{2}$ across all countries, while the random effects ${\alpha }_{c}$ and ${\beta }_{c}$ were estimated separately for each. For a detailed description of this method see Sera, Armstrong (Sera et al. 2019).

### Cannabis use following decriminalization in the Swedish setting

Using the parameters described above we predicted cannabis use in Sweden assuming that recreational cannabis use was decriminalized in 2017. The year was chosen arbitrary to display both prior and post decriminalization trends during the last 20 years. The variables to be incorporated into the model consisted of:

Year: Time since start of the time series, *X*: macro level covariate, *Intervention*: a binary variable set to 0 prior to the intervention and to 1 post intervention, *Time post intervention*: years after intervention was implemented.

### Evaluating the models

The model fits of the different macro level factors were compared with the Bayesian Information Criteria (BIC) for each estimated model. In addition, graphical presentations were used to visually inspect differences on the outcome.

## Results

Country-specific characteristics (year of decriminalization, means and values at year of decriminalization of the macro factors) are presented in Table 2. The year of decriminalization ranged from 2001 in Luxemburg and Portugal to 2022 in Italy. The GINI-coefficients varied for 0·25 in Slovenia up to 0·4–0·5 among various states of US. SDI was lowest in Portugal (0·70) and highest in Switzerland (0·91). The US had the highest share of individuals with tertiary education (0·40), while Portugal the lowest (0·15), here no data for Australia was found. We observed only minor variation in the share of population in young adulthood (0·11 to 0·14). The GDP per capita was highest in Luxemburg ($92 906) and lowest in Croatia ($11 672). Sweden was not an outlier concerning these measures that are located around the average for most factors, except for the GINI-coefficient (being on the lower end of the distribution) and SDI (higher end). This indicates that Sweden was the most egalitarian country among those included in the analysis.

**Table 2:** Description of the included countries. Means and value at year of decriminalization in parentheses

**Country**	Year of decriminalization	Gini-coefficient	SDI	Share with tertiary education	Share age 15–24 years old	GDP per capita
Australia	2020	0·34 (0·34)	0·80 (0·84)	-	0·14 (0·12)	$44 286 ($53 250)
Austria	2016	0·30 (0·31)	0·82 (0·84)	0·20 (0·28)	0·12 (0·12)	$41 769 ($45 279)
Belgium	2003	0·28 (0·28)	0·81 (0·79)	0·30 (0·24)	0·12 (0·12)	$39 210 ($30 708)
Croatia	2013	0·32 (0·32)	0·75 (0·77)	0·18 (0·21)	0·12 (0·11)	$11 672 ($14 002)
Czech Republic	2013	0·26 (0·26)	0·80 (0·82)	0·17 (0·18)	0·13 (0·10)	$16 728 ($20 257)
Estonia	2005	0·34 (0·27)	0·79 (0·77)	0·34 (0·32)	0·13 (0·15)	$14 773 ($10 429)
Italy	2022	0·35 (0·32)	0·77 (0·81)	0·13 (0·17)	0·11 (0·10)	$30 989 ($35 654)
Luxemburg	2001	0·32 (0·31)	0·85 (0·83)	0·29 (0·16)	0·11 (0·11)	$92 906 ($48 719)
Portugal	2001	0·37 (0·39)	0·70 (0·67)	0·15 (0·08)	0·12 (0·15)	$19 196 ($11 735)
Slovenia	2013	0·25 (0·26)	0·80 (0·83)	0·21 (0·22)	0·12 (0·10)	$20 152 ($23 250)
Spain	2017	0·35 (0·35)	0·73 (0·76)	0·27 (0·31)	0·12 (0·09)	$25 423 ($28 395)
Switzerland	2012	0·33 (0·32)	0·91 (0·92)	0·31 (0·31)	0·11 (0·12)	$69 353 ($86 304)
United States				0·40	0·14	$50 114
Hawaii	2020	0·44 (0·46)	0·83 (0·87)	*	*	*
Louisiana	2021	0·49 (0·50)	0·78 (0·83)	*	*	*
New Hampshire	2017	0·43 (0·44)	0·87 (0·89)	*	*	*
North Dakota	2019	0·45 (0·46)	0·83 (0·87)	*	*	*
Sweden		0·28	0·86	0·29	0·12	$45 899

Data include all adults (age 16 and above)

^*^) Only data on country-level was observed

### Predicted cannabis use following decriminalization

In Table 3, the estimated fixed effects parameters are presented separately for each macro factor as well as for the estimated model only depending on time. These coefficients are presented both for past 12-month and past 30-day prevalence separately.

**Table 3:** Estimated parameters for predicting prevalence of cannabis use after decriminalization, fixed effects

	**Time [95% CI]**	**Interaction between Time and Gini-coefficient [95% CI]**	**Interaction between Time and SDI [95% CI]**	**Interaction between Time and Share with tertiary education [95% CI]**	**Interaction between Time and Share age 15–24 years old [95% CI]**	**Interaction between Time and GDP per capita [95% CI]**
Past 12-month						
Initial change in cannabis use directly after decriminalization	0·946 [0·686; 1·205]	3·002 [2·229; 3·775]	1·175 [0·856; 1·494]	3·221 [2·320; 4·122]	8·554 [6·201; 10·907]	0·187 [0·133; 0·240]
Time-dependent change since decriminalization	−0·053 [−0·104; −0·001]	−0·151 [−0·312;0·009]	−0·045 [−0·105;0·015]	−0·170 [−0·305; −0·035]	−0·226 [−0·728; 0·275]	0·004 [−0·004; 0·013]
Past 30-day						
Initial change in cannabis use directly after decriminalization	0·302 [0·074; 0·529]	1·088 [0·406; 1·771]	0·401 [0·118; 0·684]	2·590 [1·798; 3·382]	2·887 [0·815; 4·958]	0·062 [0·009; 0·116]
Time-dependent change since decriminalization	0·055 [0·010; 0·101]	0·144 [0·008; 0·279]	0·081 [0·029; 0·133]	0·223 [0·109; 0·337]	0·587 [0·148; 1·027]	0·012 [0·005; 0·019]

*CI* Confidence interval

Including Australia in all results of past 12-month use except share with tertiary education

GDP per capita scaled to per ten thousand dollar

Data include all adults (age 16 and above)

The parameter indicating the initial change in cannabis use directly after decriminalization showed a consistently significant increase for both past 12-month and past 30-day use. This increase was approximately 3 times higher for past 12-month use compared to past 30-day use (0·946 vs. 0·302 without macro-factors), except for the model including tertiary education, where the estimated initial increase was similar for past 12-month and past 30-day use.

For the time-dependent change post decriminalization, the estimated parameters for past 12-month and past 30-day prevalences were in opposite directions. For the past 12-month cannabis use, the estimates generally revealed a reduction of the slope over time, while the estimates for the past 30-day displayed a significant increase of the slope over time.

The evaluation of model fit is reported in table A2 in Appendix A1. The BIC for the model fit were very similar for all models predicting past 12-month cannabis use, while the model containing the proportion high educational attainment had the best fit (lowest BIC) in the prediction of past 30-day use.

In a graphical presentation of the predicted outcomes for each country together with the observed outcomes (see Figures A1 and A2 in Appendix A1 for past 12-month and for past 30-day prevalences) most predictions seemed to fit the data well.

In general, all models produced fairly consistent results, indicating that the prediction was not dependent on the choice of the macro-factor. As additional sensitivity analysis, all predictive models were run, excluding the U.S. states, given the potential difference in policy implementation between national and sub-national jurisdictions. The predictions with and without U.S. states displayed no appreciable differences. To understand whether the choice of the year for decriminalization had no direct effect on the results we run sensitivity analyses with 2015 or 2018 as the year of decriminalization, yielding similar results as for 2017.

**Fig. 1:**
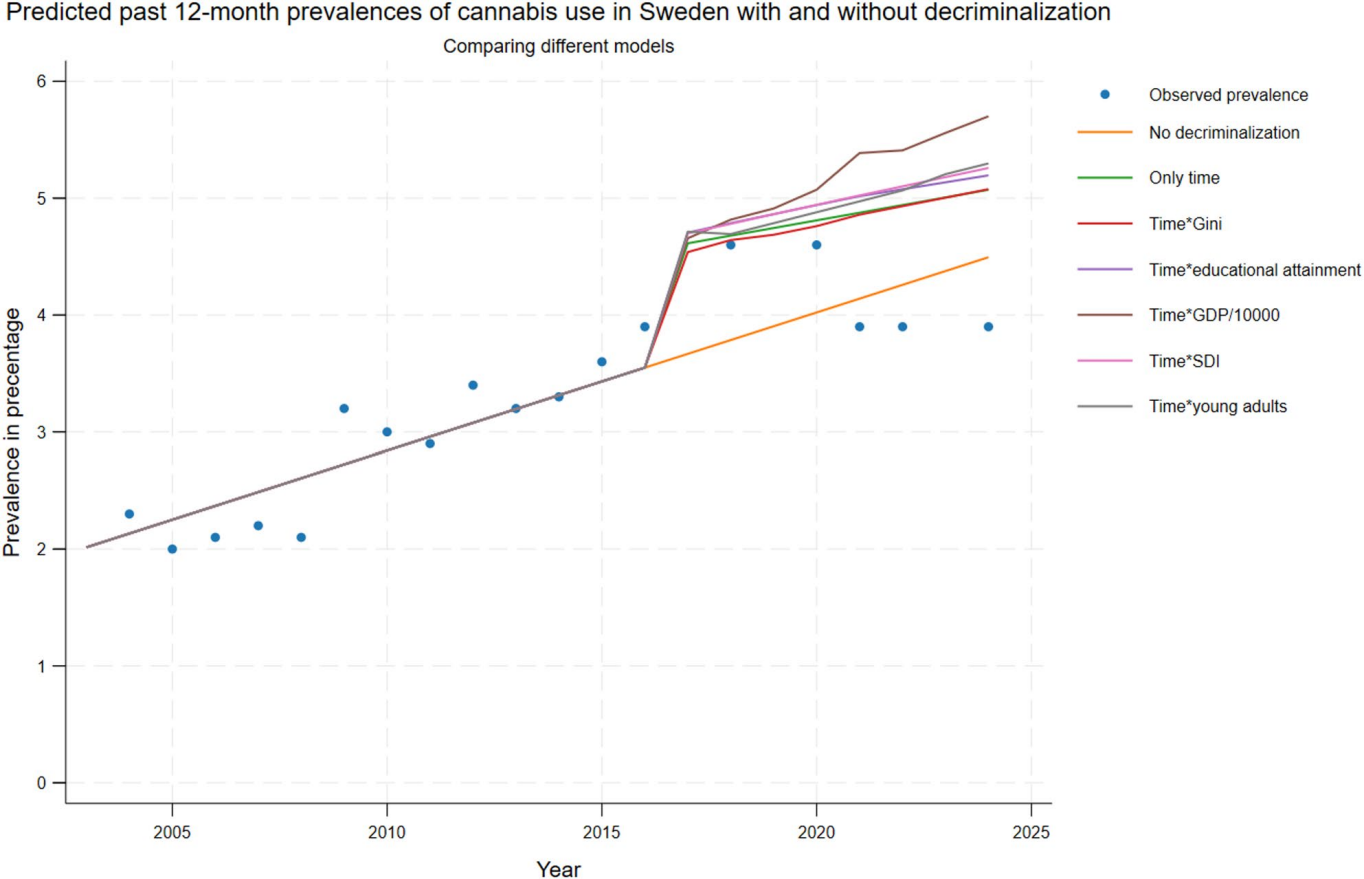
Predicted past 12-month prevalences of cannabis use in Sweden pre and post decriminalization for all adults (age 16 and above). For detail see section “Model definition”

Figure 1 (past 12-month use) and 2 (past 30-day use) presents the predicted outcomes in Sweden, after hypothetical decriminalization of cannabis use in 2017, together with predicted outcomes without decriminalization and true observed prevalences.

Following decriminalization, a short-term increase of past 12-month cannabis use was observed (Fig. 1) of approximately 1 percentage point compared with the estimated prevalence without decriminalization. This initial increase was followed by a decreasing trend, reducing the difference between the predicted prevalence with and without decriminalization.

**Fig. 2:**
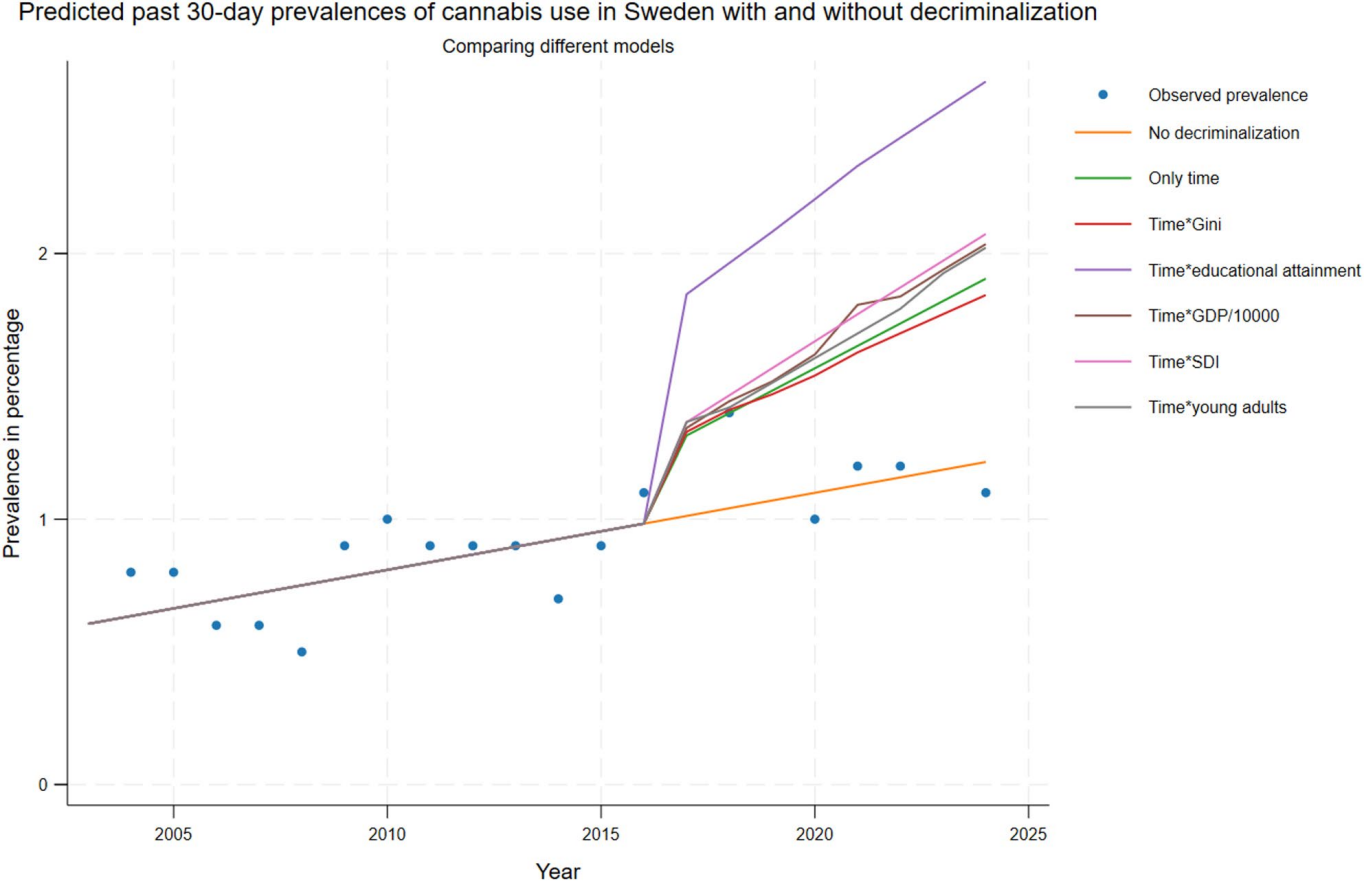
Predicted past 30-day observed prevalences of cannabis use in Sweden pre and post decriminalization for all adults (age 16 and above). For detail see section “Model definition”

After decriminalization all models predicted an initial increase of past 30-day use of cannabis (Fig. 2) of about 0.5 percentage points, indeed higher when including the share of highly educated population as macro-factor. The predicted trend after decriminalization showed a sustained increase compared to that without decriminalization widening the gap over time.

## Discussion

In this study we compared the predicted population prevalence of cannabis use in Sweden in absence and in presence of a hypothetical decriminalization of recreational cannabis use.

We found a consistent initial increase in the use of cannabis post decriminalization. This increase was evident for both past 12-month prevalence (that may be conceived as an indicator of experimental behavior) and past 30-day prevalence (more likely to reflect recurrent or regular behavior). Unpublished results from a Swedish survey support this interpretation, with much higher self-reported consumption among 30-day users compared to 12-month users (CAN 2017) (personal communication with Mats Ramstedt). The surge of prevalence close to the shift to more lenient legislation may indicate a rise of experimental use in the susceptible population when the risk of legal consequences is abolished. However, this initial increase could partly be attributed to individuals’ increased propensity to report a behavior no longer labeled as criminal. Indeed, this interpretation is supported by a previous Swedish study, that found a substantially higher level of self-reported use among individuals answering a survey where complete anonymity was secured compared to those answering a traditional survey (Andersson et al. 2023).

However, the time-dependent change in prevalence after decriminalization seemed to differ between past 12-month and past 30-day use. In fact, after the early surge the former indicator, while still increasing, tended to converge with the frequency expected in absence of the change in legislation, while the trends of past-30-day use continued to increase. If we take as a face-value, the interpretation of these two measures (experimental vs recurrent-regular use) these different trends may indicate an exhaustion over time of the pool of individuals who are just interested to try the drug. In contrast, the pool of individuals who are susceptible to transition to more advanced profiles of use would continue to increase along with the increase in trials, and possibly reach a saturation point much later. However, in absence of more refined information on actual frequency of use more complex explanations are possible, given that strictly speaking past 30-day use is just an indicator of recency of use. In addition, the misclassification of self-reports based on recalls over 12 months may be stronger than when recalls are based on a shorter time frame.

There are multiple rationales to only include policy changes past 2000. First, the absence of continuous, nationally representative population surveys on cannabis use prior to 2000 precludes a reliable evaluation of trends during earlier periods. This includes the unification of data on drug use in Europe by EUDA (previously EMCDDA) in the early 2000 s (EUDA 2026). As a matter of fact, among the countries included in this study only Belgium, Estonia, Luxembourg and Spain reported measurements on cannabis use between 1994 and 1999. Additionally, very few studies on the impact of decriminalization of drug use were published prior to 2000. In a systematic review by Scheim, Maghsoudi (Scheim et al. 2020) only 4 out of 114 included studies were published between 1970 and 1999. Moreover, given the study’s aim to estimate the effects of a potential contemporary decriminalization in Sweden, it is methodologically justified to rely on the most recent available data, as these more accurately reflect current patterns of use, the societal context, and relevant policy conditions.

To account for the cultural and demographic differences between Sweden and the countries that provided data for the prediction model, we let the effect of decriminalization vary depending on 5 country-level indicators, the GINI-coefficient, the Socio-demographic index, the share of the population with tertiary education, the share of the population age 15–24, and the gross domestic product per capita. When we compared models with and without these factors there were only minor differences in the results. One explanation may be that modeling the individual country level prevalences as random effects contributed to removal of most of the variance associated with socio-economic conditions. In addition, the chosen macro-level factors may be too broad to capture potential variation in response to decriminalization. The only macro-factor showing an impact on the results was population educational attainment, when predicting past 30-day use, that showed higher prevalences post decriminalization when adjusted for this factor. This model also exhibited better model fit (Appendix A1, table 2). One reason for this finding could be that the education varied among countries more substantially than other factors. Alternatively, this could represent different levels of risk appraisal among individuals with different levels of education. For instance, people with higher educational level might be more willing to change their behavior if there was no risk of legal consequences.

A meta-analysis of previous studies on the association between change in legislation and cannabis use among young adults by Melchior, Nakamura (Melchior et al. 2019) included thirteen studies that dealt with decriminalization of recreational use. Their analysis found no overall association between decriminalization and prevalence of cannabis use. However, direct comparison with the results in the present analysis is difficult because of the different population’s age (young adults in the meta-analysis vs. adults 16 years and older in the present sample). The five studies based on past 12-month use pre and post decriminalization showed results in line with the present study. Similar patterns were seen as well among 30-day use in four included papers. A study only including European countries, partly based on the same data as in this study, did not find significant change in prevalence of use following decriminalization (Gabri et al. 2022). However, it is worth noting that this study included fewer countries than the current one, leading to lower precision of the estimates.

This study has several methodological strengths. First, we could include more countries and states compared to earlier studies, due to the increasing tendency to decriminalization worldwide. Second, we used meta regression, treating each measurement as a specific study, also taking into account the sizes of the samples from which the prevalence estimates were derived, which increased the power of the study. Third, we included the trend post decriminalization into our model, which was not done in other studies. We also applied a multilevel modeling procedure, treating country-specific baseline cannabis prevalence as random effects.

Notwithstanding, this study also faces several limitations. The information on cannabis use was based on aggregated data, originating from different surveys across all countries. The quality of these surveys might vary substantially between countries, as well as the constructs used in the surveys, the selection of the participants and how often the surveys were conducted. We based the predictions in Sweden on estimated models rather than actual data. Finally, we needed to exclude one country (Australia) from the analysis of past 30-day use, since the corresponding information was not available.

## Conclusions

In conclusion, this study reports a country-specific prediction of population trends in cannabis use following its hypothetical decriminalization. A decriminalization of cannabis use in Sweden could lead to an initial increase of experimental use and a more sustained increase of recurrent use. Following this initial surge, experimental use tends to stabilize, while recurrent use may continue to rise, potentially reflecting an expanding pool of individuals transitioning towards dependency.

Trends in prevalence is only part of the information that should be considered when issuing new legislations regarding recreational cannabis use. Factors such as changes in health care expenditures, criminal justice cost, gang crime, social exclusion and access to care among users should also be considered.

The method we propose to predict population trends of cannabis use following its decriminalization may easily be replicated in other contexts and be used to facilitate decision-making about policy changes. It can also provide information on potential health consequences of legislative changes from a stricter to a more lenient policy on cannabis use. Given the recent debate regarding the governmental drug policy, implying the need for drug policy evaluation in Sweden this study provides information that is directly relevant for the issue at stake. In fact, knowledge of a probable scenario concerning cannabis use in the population following a more lenient policy may lead to a more informed debate and facilitate policy development and evaluation. This is not an unlikely future scenario, given the changes that have happened in other western countries during the last 10 years and the increasing demand for decriminalization from the public in Sweden (Stenström et al. 2024), even though being a country with historically strong anti-drug norms.

## Supplementary Information

Supplementary Material 1.

Supplementary Material 2.

Supplementary Material 3.

Supplementary Material 4.

## Data Availability

The dataset supporting the conclusions of this article is included within the article and its additional files.
