# Supplementary material for: Expected population prevalence following decriminalization of recreational use of cannabis in Sweden
Source: J Cannabis Res. 2026 Feb 13;8:32. doi: 10.1186/s42238-026-00405-z (PMC12930985; doi:10.1186/s42238-026-00405-z)
Supplement: Supplementary file 1 — Supplementary Material 1. [file 42238_2026_405_MOESM1_ESM.docx]

# Supplementary material for: Expected population outcomes following decriminalization of recreational use of cannabis in Sweden

Filip Andersson, MSc^1,2^, Robert Thiesmeier, MSc^1^, Cecilia Magnusson, PhD^1,2^, Nicola Orsini, PhD^1,2^, Mats Ramstedt, PhD^3,4^, Maria Rosaria Galanti, PhD^1^

^1^Department of Global Public Health, Karolinska Institutet, SE-17177 Stockholm, Sweden; ^2^Centre for Epidemiology and Community Medicine, Stockholm Health Care District, Stockholm Region, Box 45436 10431 Stockholm, Sweden; ^3^Department of Clinical Neuroscience, Karolinska Institutet, SE-17177 Stockholm, Sweden; ^4^The Swedish Council for Information on Alcohol and Other Drugs, Östergötagatan 90 11664 Stockholm, Sweden

# Appendix A1

Table A1 Description of the data sources.

| **Variable** | **Country** | **Source** |
| --- | --- | --- |
| Socio-Demographic index | All | Global Burden of Disease Collaborative Network. Global Burden of Disease Study 2021 (GBD 2021) Socio-Demographic Index (SDI) 1950–2021. Seattle, United States of America: Institute for Health Metrics and Evaluation (IHME), 2024. |
| Gini-coefficient | All – only country level | Gini Coefficient World Bank |
| Gini-coefficient | Hawaii  Louisiana  New Hampshire  North Dakota | [Useful Stats: Income inequality across the states \| SSTI](https://ssti.org/blog/useful-stats-income-inequality-across-states) |
| Share with tertiary education | All – only country level, except Australia | https://w3.unece.org/PXWeb2015/pxweb/en/STAT/ |
| Share age 15-24 years old | All – only country level | [All countries compared for People > Age distribution > Population aged 15-24 > Percent](https://www.nationmaster.com/country-info/stats/People/Age-distribution/Population-aged-15--24/Percent#1995) |
| GDP per capita | All – only country level | https://countryeconomy.com/gdp |
| Cannabis use | Australia | National Drug Strategy Household Survey 2022–2023: Cannabis in the NDSHS - Australian Institute of Health and Welfare |
| Cannabis use | Austria, Belgium, Croatia, Czech Republic, Estonia, Italy, Luxemburg, Portugal, Slovenia, Spain, Sweden | EMCDDA – Statistical Bulletin 2004-2024 |
| Cannabis use | Switzerland | [Cannabis consumption (age: 15-64) \| MonAM \| FOPH](https://ind.obsan.admin.ch/en/indicator/monam/cannabis-consumption-age-15-64) |
| Cannabis use | Hawaii, Louisiana, New Hampshire, North Dakota | [National Survey on Drug Use and Health](https://www.samhsa.gov/data/data-we-collect/nsduh-national-survey-drug-use-and-health) |
| Share with tertiary education | Croatia – added data | [Statistics \| Eurostat](https://ec.europa.eu/eurostat/databrowser/view/edat_lfs_9903/default/table?lang=en) |

Table A2 BIC of the estimated models of cannabis use after decriminalization.

|  | **Time**  **[95% CI]** | **Interaction between Time and Gini-coefficient [95% CI]** | **Interaction between Time and SDI**  **[95% CI]** | **Interaction between Time and Share with tertiary education**  **[95% CI]** | **Interaction between Time and Share age 15-24 years old**  **[95% CI]** | **Interaction between Time and GDP per capita**  **[95% CI]** |
| --- | --- | --- | --- | --- | --- | --- |
| **Past 12-month** |  |  |  |  |  |  |
| BIC | 1738·953 | 1732·732 | 1738·556 | 1739·994 | 1710·781 | 1744·510 |
| **Past 30-days** |  |  |  |  |  |  |
| BIC | -11·824 | -13·795 | -15·042 | -129·993 | -12·496 | -12·914 |


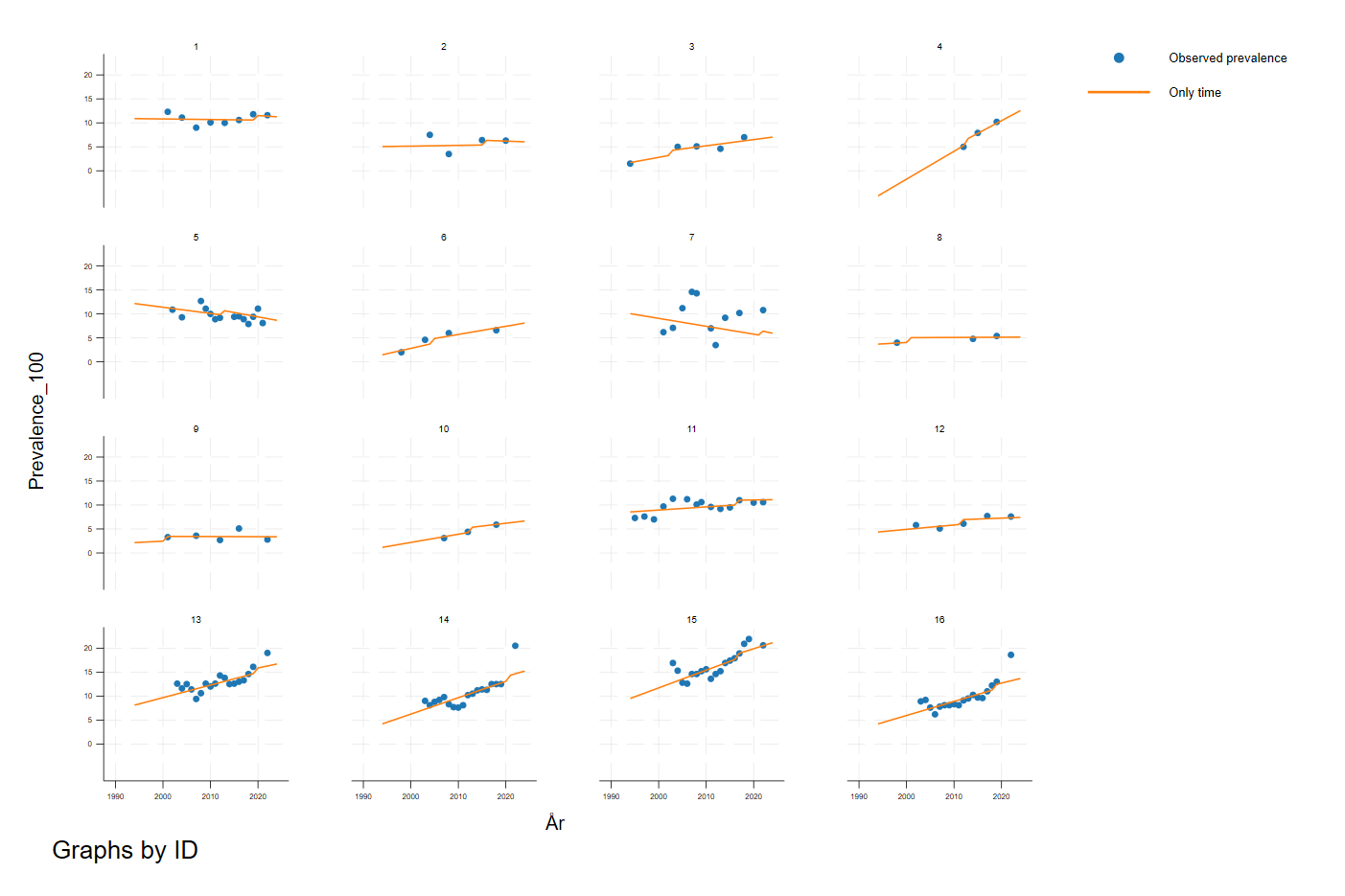


Figure A1 Predicted past 12-month prevalence of cannabis use pre and post decriminalization, by country.


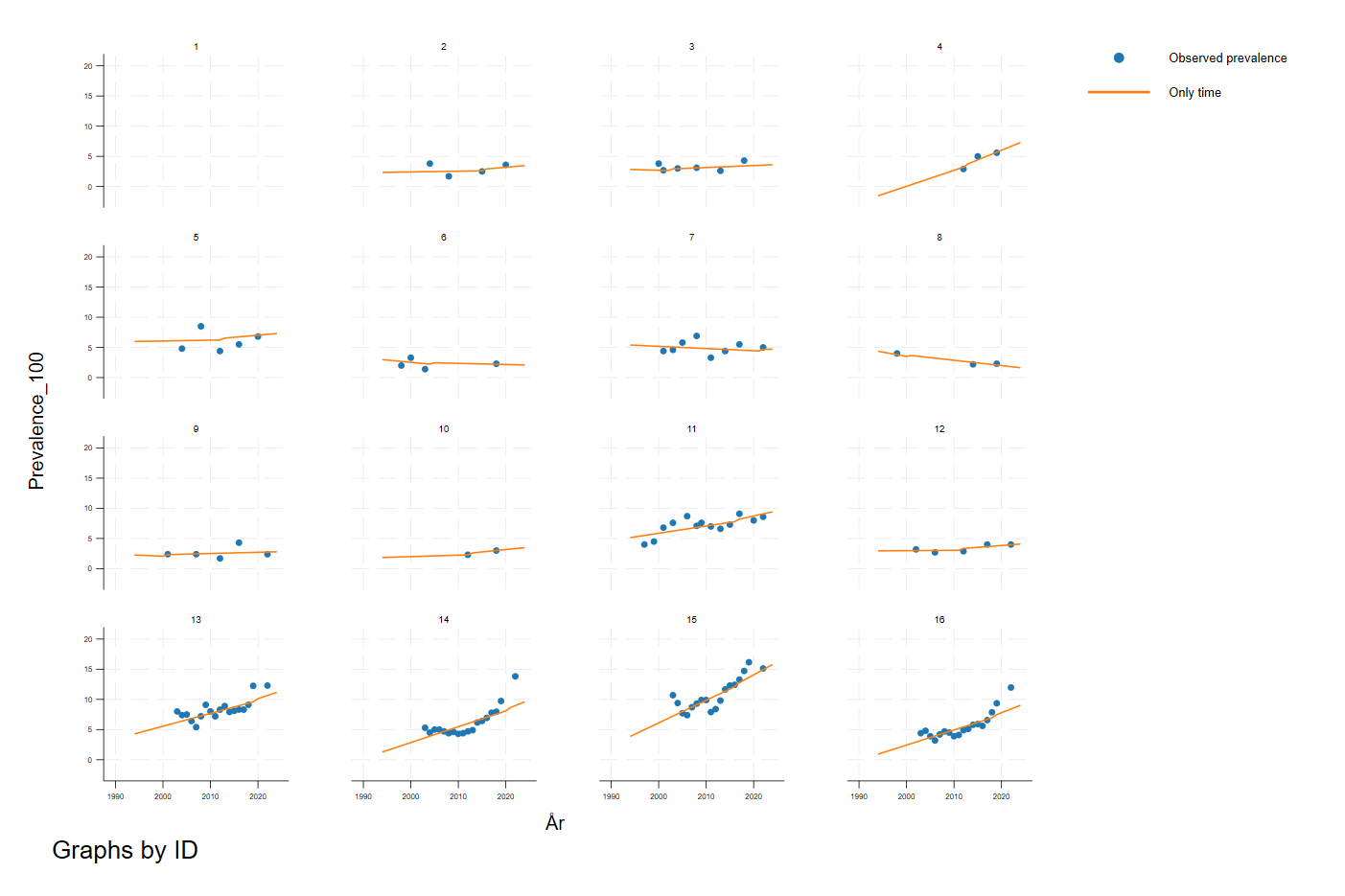


Figure A2 Predicted post 30-days prevalences of cannabis use pre and post decriminalization.
