# Supplementary material for: Expected population prevalence following decriminalization of recreational use of cannabis in Sweden
Source: J Cannabis Res. 2026 Feb 13;8:32. doi: 10.1186/s42238-026-00405-z (PMC12930985; doi:10.1186/s42238-026-00405-z)
Supplement: Supplementary file 3 — Supplementary Material 3. [file 42238_2026_405_MOESM3_ESM.docx]

# Supplementary material for: Expected population outcomes following decriminalization of recreational use of cannabis in Sweden

Filip Andersson, MSc^1,2^, Robert Thiesmeier, MSc^1^, Cecilia Magnusson, PhD^1,2^, Nicola Orsini, PhD^1,2^, Mats Ramstedt, PhD^3,4^, Maria Rosaria Galanti, PhD^1^

^1^Department of Global Public Health, Karolinska Institutet, SE-17177 Stockholm, Sweden; ^2^Centre for Epidemiology and Community Medicine, Stockholm Health Care District, Stockholm Region, Box 45436 10431 Stockholm, Sweden; ^3^Department of Clinical Neuroscience, Karolinska Institutet, SE-17177 Stockholm, Sweden; ^4^The Swedish Council for Information on Alcohol and Other Drugs, Östergötagatan 90 11664 Stockholm, Sweden

Appendix A3

# **Stata codes**

## Yearly cannabis use

///Decriminalization effect

clear

import excel “Appendix A2.xlsx", sheet("Data") firstrow

///drop if ID==1

///drop if ID==16 | ID==15 | ID==14 | ID==13 ///Without US

set cformat %9.3f

gen time = T0int*Xt

gen N_cannabis = Prevalence*N

destring educ, replace force

gen educ_prec = educ/100

rename _24 young_adults

replace young_adults = subinstr(young_adults,"%", "",.)

destring young_adults, replace force

gen young_adults_prec = young_adults/100

drop educ

drop young_adults

rename educ_prec educ

rename young_adults_prec young_adults

gen GDP_1000 = GDP/1000

gen GDP_10000 = GDP/10000

meta esize N_cannabis N, esize(prop) random

sort Country Year

xtset ID Year, yearly

///// Predefined model: a+b1*year+b2*intervention+b3*time_since_intervention

replace Xt = 0 if Country=="Sweden" & Year>2016

replace time = T0int*Xt if Country=="Sweden" & Year>2016

meta meregress Prevalence c.Xt c.time, noconstant || Country:year , essevariable(_meta_se)

estimates store EY1

lincom Xt*100

lincom time*100

predict Phat1re1 Phat1re2, reffects

replace Xt = 1 if Country=="Sweden" & Year>2016

replace time = T0int*Xt if Country=="Sweden" & Year>2016

predict Phat1fe, xb

predict Phat1stdp1, stdp

gen Phat1 = Phat1re1*year+Phat1re2+Phat1fe

generate Phat1lb_100 = (Phat1 - invnormal(0.975)*Phat1stdp1)*100

generate Phat1ub_100 = (Phat1 + invnormal(0.975)*Phat1stdp1)*100

gen Phat0 = Phat1re1*year+Phat1re2

gen Phat1_100 = Phat1*100

gen Phat0_100 = Phat0*100

gen Prevalence_100 = Prevalence*100

tw (scatter Prevalence_100 Year if Country=="Sweden" & Year>2002) (line Phat1_100 Year if Country=="Sweden" & Year>2002) (line Phat1lb_100 Year if Country=="Sweden" & Year>2002) (line Phat1ub_100 Year if Country=="Sweden" & Year>2002) (line Phat0_100 Year if Country=="Sweden" & Year>2002), yscale(range(0,6))

///// Predefined model: a+b1*year+b2*intervention*gini+b3*time_since_intervention*gini

replace Xt = 0 if Country=="Sweden" & Year>2016

replace time = T0int*Xt if Country=="Sweden" & Year>2016

meta meregress Prevalence c.Xt#c.GINI c.time#c.GINI , noconstant || Country:year , essevariable(_meta_se)

estimates store EY2

lincom c.Xt#c.GINI*100

lincom c.time#c.GINI*100

predict Phat2re1 Phat2re2, reffects

replace Xt = 1 if Country=="Sweden" & Year>2016

replace time = T0int*Xt if Country=="Sweden" & Year>2016

predict Phat2fe, xb

gen Phat2 = Phat2re1*year+Phat2re2+Phat2fe

gen Phat2_100 = Phat2*100

tw (scatter Prevalence_100 Year if Country=="Sweden" & Year>2002) (line Phat2_100 Year if Country=="Sweden" & Year>2002) (line Phat0_100 Year if Country=="Sweden" & Year>2002), yscale(range(0,6))

///// Predefined model: a+b1*year+b2*intervention*educ+b3*time_since_intervention*educ

replace Xt = 0 if Country=="Sweden" & Year>2016

replace time = T0int*Xt if Country=="Sweden" & Year>2016

meta meregress Prevalence c.Xt#c.educ c.time#c.educ , noconstant || Country:year , essevariable(_meta_se)

estimates store EY3

lincom c.Xt#c.educ*100

lincom c.time#c.educ*100

predict Phat3re1 Phat3re2, reffects

replace Xt = 1 if Country=="Sweden" & Year>2016

replace time = T0int*Xt if Country=="Sweden" & Year>2016

predict Phat3fe, xb

gen Phat3 = Phat3re1*year+Phat3re2+Phat3fe

gen Phat3_100 = Phat3*100

tw (scatter Prevalence_100 Year if Country=="Sweden" & Year>2002) (line Phat3_100 Year if Country=="Sweden" & Year>2002) (line Phat0_100 Year if Country=="Sweden" & Year>2002), yscale(range(0,6))

///// Predefined model: a+b1*year+b2*intervention*gdp+b3*time_since_intervention*gdp

replace Xt = 0 if Country=="Sweden" & Year>2016

replace time = T0int*Xt if Country=="Sweden" & Year>2016

//meta meregress Prevalence c.Xt#c.GDP_1000 c.time#c.GDP_1000 , noconstant || Country:year , essevariable(_meta_se)

meta meregress Prevalence c.Xt#c.GDP_10000 c.time#c.GDP_10000 , noconstant || Country:year , essevariable(_meta_se)

estimates store EY4

lincom c.Xt#c.GDP_10000*100

lincom c.time#c.GDP_10000*100

predict Phat4re1 Phat4re2, reffects

replace Xt = 1 if Country=="Sweden" & Year>2016

replace time = T0int*Xt if Country=="Sweden" & Year>2016

predict Phat4fe, xb

gen Phat4 = Phat4re1*year+Phat4re2+Phat4fe

gen Phat4_100 = Phat4*100

tw (scatter Prevalence_100 Year if Country=="Sweden" & Year>2002) (line Phat4_100 Year if Country=="Sweden" & Year>2002) (line Phat0_100 Year if Country=="Sweden" & Year>2002), yscale(range(0,6))

///// Predefined model: a+b1*year+b2*intervention*SDI+b3*time_since_intervention*SDI

replace Xt = 0 if Country=="Sweden" & Year>2016

replace time = T0int*Xt if Country=="Sweden" & Year>2016

meta meregress Prevalence c.Xt#c.SDI c.time#c.SDI , noconstant || Country:year , essevariable(_meta_se)

estimates store EY5

lincom c.Xt#c.SDI*100

lincom c.time#c.SDI*100

predict Phat5re1 Phat5re2, reffects

replace Xt = 1 if Country=="Sweden" & Year>2016

replace time = T0int*Xt if Country=="Sweden" & Year>2016

predict Phat5fe, xb

gen Phat5 = Phat5re1*year+Phat5re2+Phat5fe

gen Phat5_100 = Phat5*100

tw (scatter Prevalence_100 Year if Country=="Sweden" & Year>2002) (line Phat5_100 Year if Country=="Sweden" & Year>2002) (line Phat0_100 Year if Country=="Sweden" & Year>2002), yscale(range(0,6))

///// Predefined model: a+b1*year+b2*intervention*age+b3*time_since_intervention*age

replace Xt = 0 if Country=="Sweden" & Year>2016

replace time = T0int*Xt if Country=="Sweden" & Year>2016

meta meregress Prevalence c.Xt#c.young_adults c.time#c.young_adults , noconstant || Country:year , essevariable(_meta_se)

estimates store EY6

predict Phat6re1 Phat6re2, reffects

lincom c.Xt#c.young_adults*100

lincom c.time#c.young_adults*100

replace Xt = 1 if Country=="Sweden" & Year>2016

replace time = T0int*Xt if Country=="Sweden" & Year>2016

predict Phat6fe, xb

gen Phat6 = Phat6re1*year+Phat6re2+Phat6fe

gen Phat6_100 = Phat6*100

tw (scatter Prevalence_100 Year if Country=="Sweden" & Year>2002) (line Phat6_100 Year if Country=="Sweden" & Year>2002) (line Phat0_100 Year if Country=="Sweden" & Year>2002), yscale(range(0,6))

estimates stats EY1 EY2 EY3 EY4 EY5 EY6, all

///////////Graphical presentations

ssc install grstyle //if grstyle not installed

grstyle init

grstyle set plain, grid

grstyle set color d3

grstyle set legend 2, nobox

grstyle set graphsize 10cm 15cm

grstyle set size 8pt: heading

grstyle set size 6pt: subheading axis_title

grstyle set size 5pt: subheading axis_title_gap

grstyle set size 4pt: tick_label key_label

grstyle set symbolsize 3 4 5, pt

grstyle set linewidth .8pt: plineplot

grstyle set linewidth .4pt: legend axisline tick major_grid

grstyle set linewidth 0: pmark

grstyle set margin 0.3cm

twoway (scatter Prevalence_100 Year if Country=="Sweden" & Year>2002, sort) (line Phat0_100 Year if Country=="Sweden" & Year>2002) (line Phat1_100 Year if Country=="Sweden" & Year>2002) (line Phat2_100 Year if Country=="Sweden" & Year>2002) (line Phat3_100 Year if Country=="Sweden" & Year>2002) (line Phat4_100 Year if Country=="Sweden" & Year>2002) (line Phat5_100 Year if Country=="Sweden" & Year>2002) (line Phat6_100 Year if Country=="Sweden" & Year>2002), title("Predicted yearly observed prevalences of cannabis use in Sweden with and without decriminalization") subtitle("Comparing different models") xtitle("Year") ytitle("Prevalence in precentage") legend(order(1 "Observed prevalence" 2 "No decriminalization" 3 "Only time" 4 "Time*Gini" 5 "Time*educational attainment" 6 "Time*GDP/10000 per capita" 7 "Time*SDI" 8 "Time*young adults")) yscale(range(0,6)) ytick(0(1)6) ylabel(0 1 2 3 4 5 6)

drop if ID==17

xtline Prevalence_100 if Country != "Sweden", recast(scatter) addplot((line Phat1_100 Year if Country != "Sweden")) ylabel(0(5)20) legend(order(1 "Observed prevalence" 2 "Only time"))

## Monthly cannabis use

///Decriminalization effect

clear

import excel “Appendix A2.xlsx", sheet("Data") firstrow

//drop if ID==1

///drop if ID==16 | ID==15 | ID==14 | ID==13 ///Without US

set cformat %9.3f

gen time = T0int*Xt

destring Share_M, replace force

gen Prevalence_M = Share_M/100

gen N_cannabis = Prevalence_M*N

destring educ, replace force

gen educ_prec = educ/100

rename _24 young_adults

replace young_adults = subinstr(young_adults,"%", "",.)

destring young_adults, replace force

gen young_adults_prec = young_adults/100

drop educ

drop young_adults

rename educ_prec educ

rename young_adults_prec young_adults

gen GDP_1000 = GDP/1000

gen GDP_10000 = GDP/10000

meta esize N_cannabis N, esize(prop) random

sort Country Year

xtset ID Year, yearly

///// Predefined model: a+b1*year+b2*intervention+b3*time_since_intervention

replace Xt = 0 if Country=="Sweden" & Year>2016

replace time = T0int*Xt if Country=="Sweden" & Year>2016

meta meregress Prevalence_M c.Xt c.time , noconstant || Country:year , essevariable(_meta_se)

estimates store EM1

lincom Xt*100

lincom time*100

predict Phat1re1 Phat1re2, reffects

replace Xt = 1 if Country=="Sweden" & Year>2016

replace time = T0int*Xt if Country=="Sweden" & Year>2016

predict Phat1fe, xb

predict Phat1stdp1, stdp

gen Phat1 = Phat1re1*year+Phat1re2+Phat1fe

generate Phat1lb_100 = (Phat1 - invnormal(0.975)*Phat1stdp1)*100

generate Phat1ub_100 = (Phat1 + invnormal(0.975)*Phat1stdp1)*100

gen Phat0 = Phat1re1*year+Phat1re2

gen Phat1_100 = Phat1*100

gen Phat0_100 = Phat0*100

gen Prevalence_100 = Prevalence_M*100

tw (scatter Prevalence_100 Year if Country=="Sweden" & Year>2002) (line Phat1_100 Year if Country=="Sweden" & Year>2002) (line Phat1lb_100 Year if Country=="Sweden" & Year>2002) (line Phat1ub_100 Year if Country=="Sweden" & Year>2002) (line Phat0_100 Year if Country=="Sweden" & Year>2002), yscale(range(0,2))

///// Predefined model: a+b1*year+b2*intervention*gini+b3*time_since_intervention*gini

replace Xt = 0 if Country=="Sweden" & Year>2016

replace time = T0int*Xt if Country=="Sweden" & Year>2016

meta meregress Prevalence_M c.Xt#c.GINI c.time#c.GINI, noconstant || Country:year , essevariable(_meta_se)

estimates store EM2

lincom c.Xt#c.GINI*100

lincom c.time#c.GINI*100

predict Phat2re1 Phat2re2, reffects

replace Xt = 1 if Country=="Sweden" & Year>2016

replace time = T0int*Xt if Country=="Sweden" & Year>2016

predict Phat2fe, xb

gen Phat2 = Phat2re1*year+Phat2re2+Phat2fe

gen Phat2_100 = Phat2*100

tw (scatter Prevalence_100 Year if Country=="Sweden" & Year>2002) (line Phat2_100 Year if Country=="Sweden" & Year>2002) (line Phat0_100 Year if Country=="Sweden" & Year>2002), yscale(range(0,2))

///// Predefined model: a+b1*year+b2*intervention*educ+b3*time_since_intervention*educ

replace Xt = 0 if Country=="Sweden" & Year>2016

replace time = T0int*Xt if Country=="Sweden" & Year>2016

meta meregress Prevalence_M c.Xt#c.educ c.time#c.educ, noconstant || Country:year , essevariable(_meta_se)

estimates store EM3

lincom c.Xt#c.educ*100

lincom c.time#c.educ*100

predict Phat3re1 Phat3re2, reffects

replace Xt = 1 if Country=="Sweden" & Year>2016

replace time = T0int*Xt if Country=="Sweden" & Year>2016

predict Phat3fe, xb

gen Phat3 = Phat3re1*year+Phat3re2+Phat3fe

gen Phat3_100 = Phat3*100

tw (scatter Prevalence_100 Year if Country=="Sweden" & Year>2002) (line Phat3_100 Year if Country=="Sweden" & Year>2002) (line Phat0_100 Year if Country=="Sweden" & Year>2002), yscale(range(0,2))

///// Predefined model: a+b1*year+b2*intervention*gdp+b3*time_since_intervention*gdp

replace Xt = 0 if Country=="Sweden" & Year>2016

replace time = T0int*Xt if Country=="Sweden" & Year>2016

meta meregress Prevalence_M c.Xt#c.GDP_10000 c.time#c.GDP_10000, noconstant || Country:year , essevariable(_meta_se)

estimates store EM4

lincom c.Xt#c.GDP_10000*100

lincom c.time#c.GDP_10000*100

predict Phat4re1 Phat4re2, reffects

replace Xt = 1 if Country=="Sweden" & Year>2016

replace time = T0int*Xt if Country=="Sweden" & Year>2016

predict Phat4fe, xb

gen Phat4 = Phat4re1*year+Phat4re2+Phat4fe

gen Phat4_100 = Phat4*100

tw (scatter Prevalence_100 Year if Country=="Sweden" & Year>2002) (line Phat4_100 Year if Country=="Sweden" & Year>2002) (line Phat0_100 Year if Country=="Sweden" & Year>2002), yscale(range(0,2))

///// Predefined model: a+b1*year+b2*intervention*SDI+b3*time_since_intervention*SDI

replace Xt = 0 if Country=="Sweden" & Year>2016

replace time = T0int*Xt if Country=="Sweden" & Year>2016

meta meregress Prevalence_M c.Xt#c.SDI c.time#c.SDI, noconstant || Country:year , essevariable(_meta_se)

estimates store EM5

lincom c.Xt#c.SDI*100

lincom c.time#c.SDI*100

predict Phat5re1 Phat5re2, reffects

replace Xt = 1 if Country=="Sweden" & Year>2016

replace time = T0int*Xt if Country=="Sweden" & Year>2016

predict Phat5fe, xb

gen Phat5 = Phat5re1*year+Phat5re2+Phat5fe

gen Phat5_100 = Phat5*100

tw (scatter Prevalence_100 Year if Country=="Sweden" & Year>2002) (line Phat5_100 Year if Country=="Sweden" & Year>2002) (line Phat0_100 Year if Country=="Sweden" & Year>2002), yscale(range(0,2))

///// Predefined model: a+b1*year+b2*intervention*age+b3*time_since_intervention*age

replace Xt = 0 if Country=="Sweden" & Year>2016

replace time = T0int*Xt if Country=="Sweden" & Year>2016

meta meregress Prevalence_M c.Xt#c.young_adults c.time#c.young_adults, noconstant || Country:year , essevariable(_meta_se)

estimates store EM6

lincom c.Xt#c.young_adults*100

lincom c.time#c.young_adults*100

predict Phat6re1 Phat6re2, reffects

replace Xt = 1 if Country=="Sweden" & Year>2016

replace time = T0int*Xt if Country=="Sweden" & Year>2016

predict Phat6fe, xb

gen Phat6 = Phat6re1*year+Phat6re2+Phat6fe

gen Phat6_100 = Phat6*100

tw (scatter Prevalence_100 Year if Country=="Sweden" & Year>2002) (line Phat6_100 Year if Country=="Sweden" & Year>2002) (line Phat0_100 Year if Country=="Sweden" & Year>2002), yscale(range(0,2))

estimates stats EM1 EM2 EM3 EM4 EM5 EM6, all

///////////Graphical presentations

grstyle init

grstyle set plain, grid

grstyle set color d3

grstyle set legend 2, nobox

grstyle set graphsize 10cm 15cm

grstyle set size 8pt: heading

grstyle set size 6pt: subheading axis_title

grstyle set size 5pt: subheading axis_title_gap

grstyle set size 4pt: tick_label key_label

grstyle set symbolsize 3 4 5, pt

grstyle set linewidth .8pt: plineplot

grstyle set linewidth .4pt: legend axisline tick major_grid

grstyle set linewidth 0: pmark

grstyle set margin 0.3cm

twoway (scatter Prevalence_100 Year if Country=="Sweden" & Year>2002, sort) (line Phat0_100 Year if Country=="Sweden" & Year>2002) (line Phat1_100 Year if Country=="Sweden" & Year>2002) (line Phat2_100 Year if Country=="Sweden" & Year>2002) (line Phat3_100 Year if Country=="Sweden" & Year>2002) (line Phat4_100 Year if Country=="Sweden" & Year>2002) (line Phat5_100 Year if Country=="Sweden" & Year>2002) (line Phat6_100 Year if Country=="Sweden" & Year>2002), title("Predicted monthly observed prevalences of cannabis use in Sweden with and without decriminalization") subtitle("Comparing different models") xtitle("Year") ytitle("Prevalence in percentage") legend(order(1 "Observed prevalence" 2 "No decriminalization" 3 "Only time" 4 "Time*Gini" 5 "Time*educational attainment" 6 "Time*GDP/10000 per capita" 7 "Time*SDI" 8 "Time*young adults")) yscale(range(0,2)) ytick(0(1)2) ylabel(0 1 2)

drop if ID==17

xtline Prevalence_100 if Country != "Sweden", recast(scatter) addplot((line Phat1_100 Year if Country != "Sweden")) ylabel(0(5)20) legend(order(1 "Observed prevalence" 2 "Only time"))
