# Supplementary material for: Expected population prevalence following decriminalization of recreational use of cannabis in Sweden
Source: J Cannabis Res. 2026 Feb 13;8:32. doi: 10.1186/s42238-026-00405-z (PMC12930985; doi:10.1186/s42238-026-00405-z)
Supplement: Supplementary file 4 — Supplementary Material 4. [file 42238_2026_405_MOESM4_ESM.docx]

## Appendix A4

### **Model definition – details**

#### Baseline

Using existing survey data, we estimated the average past 12-month and past 30-day change in prevalence of cannabis use among all adults (16 years and above) over time in a time dependent linear model, assuming this change to be country-specific and independent of decriminalization. We specified the parameters as follows:

$$f\left( prevalence cannabis | year, country \right)=\alpha_{c}+\beta_{c}*year$$

The relevant parameters are, 1) $\alpha_{c}$, the prevalence of cannabis use in the specific country c at the start of the time series and 2) $\beta_{c}$, the average yearly change in the prevalence of cannabis use in that country from the start of the time series.

#### Intervention’s effect - Interrupted Time Series

Next, we defined the model to estimate the effect of decriminalization on the prevalence of cannabis use. We used an interrupted time series design, allowing for an initial effect and for a time dependent effect. This latter effect was assumed to be constant over all countries included in the study. We specified the parameters as follows:

$$f\left( change in prevalence | intervention, time post intervention \right)=m+\gamma*time$$

Thus, the relevant parameters to be estimated are 1) $m$, the average initial intervention effect, and 2) $\gamma$, the average yearly change in prevalence post intervention, where time is defined as years from the introduction of the intervention.

#### Influence of macro factors on the intervention’s effect

To consider the cultural and demographic differences between countries potentially leading to different responses to decriminalization we additionally modeled the variability in the effect of the intervention, depending on macro-level factors. An interaction term was added between the intervention effects and each of the macro level factors, one at a time. The parameters were defined as follows:

$$f\left( m+\gamma| macro level factor=X \right)=m\left( X \right)+\gamma\left( X \right)=\Delta_{1}*X+\Delta_{2}*X$$

We defined $\Delta_{1}$ to be the initial intervention effect dependent on macro level factor X, and $\Delta_{2}$ to be the average yearly change after intervention, depending on macro level factor X. For the model where we did not use a macro level factor, X is set to 1. This means that in this specific case $\Delta_{1}*X+\Delta_{2}*X= m+\gamma$, assuming independency of macro factors.
